# TWEAK/Fn14 system and crescent formation in IgA nephropathy

**DOI:** 10.1186/s12882-015-0022-8

**Published:** 2015-03-14

**Authors:** Yohei Sasaki, Yoshio Shimizu, Yusuke Suzuki, Satoshi Horikoshi, Yasuhiko Tomino

**Affiliations:** Division of Nephrology, Department of Internal Medicine, Juntendo University Faculty of Medicine, 2-1-1, Hongo, Bunkyo-ku Tokyo, 113-8421 Japan

**Keywords:** TWEAK, IgA nephropathy, Crescent formation, Proteinuria

## Abstract

**Background:**

The TNF-like weak inducer of apoptosis (TWEAK) contributes to kidney inflammation producing secretion by renal cells. The present study examined whether the level of TWEAK is associated with histologic findings in patients with IgA nephropathy (IgAN).

**Methods:**

The levels of urinary TWEAK (uTWEAK) from 116 IgAN patients, 50 non-IgA kidney disease patients, and 50 healthy individuals were measured by ELISA. Histological findings of renal biopsy specimens of patients with IgAN were evaluated according to the Oxford classification and histological classification for IgAN in Japan. We investigated the expression of TWEAK/Fn14 in renal tissues of those patients and assessed the effect of TWEAK in glomerular mesangial cells and podocytes.

**Results:**

The levels of uTWEAK in the patients with IgAN and other renal diseases were significantly higher than in the healthy controls (*P* < 0.001). In the IgAN patients, the levels of uTWEAK correlated significantly with urinary protein excretion and extracapillary proliferation (r = 0.54, *P* < 0.001 and r = 0.32, *P* < 0.001, respectively). In a comparison of the levels of uTWEAK at diagnosis with that of follow-up, the levels of uTWEAK in patients with clinical and partial remission decreased significantly. We showed not only increased expression of both TWEAK and Fn14 in IgAN patients with glomerular crescents but also TWEAK-induced cell motility in podocytes.

**Conclusions:**

The relationship between the levels of uTWEAK and clinicopathological findings observed in this study suggests that TWEAK/Fn14 system affects crescent formation and proteinuria in patients with IgAN.

## Background

The TNF-like weak inducer of apoptosis (TWEAK, TNFSF12), a TNF superfamily member, is synthesized as a type II transmembrane glycoprotein that circulates in plasma as a soluble form [1]. TWEAK is widely expressed in many cells and tissues including monocytes/macrophages, the heart, the brain, and kidneys [2]. The binding of TWEAK to its receptor, fibroblast growth factor-inducible-Fn14 (Fn14) [3,4], regulates cellular proliferation, differentiation, migration, inflammation, and apoptosis [5]. The plasma levels of soluble TWEAK have been reported to be associated with the aggravation of the endothelial function and mortality risk [6,7]. Recent studies have further indicated that the levels of urinary TWEAK (uTWEAK) correlated positively with lupus nephritis activity [8,9]. These findings suggest that TWEAK expression may reflect kidney inflammation and is associated with chronic kidney disease (CKD).

IgA nephropathy (IgAN) is the most common form of primary glomerulonephritis worldwide [10] and is one of the leading causes of end-stage renal disease (ESRD) [11]. The histologic features of IgAN show an increase in mesangial proliferation with matrix expansion; other glomerular lesions may include focal necrosis, segmental sclerosis, and crescent formations [12]. Podocyte injury is a common denominator in many forms of human glomerular diseases [13] and is characteristic of proteinuric kidney diseases, including IgAN [14]. Notably, podocyte loss from the glomerular basement membrane (GBM) in IgAN may cause the progression of proteinuria and glomerulosclerosis [15,16].

Previously, Fn14 expression was observed in tubular cells, glomerular mesangial cells, and podocytes [17,18]. TWEAK induces the expression of inflammatory cytokines, such as monocyte chemoattractant protein-1 (MCP-1), interleukin (IL)-6, RANTES, and CXCL16, and downregulates the expression of Klotho [19-22]. Therefore, TWEAK has proinflammatory effects on glomerular mesangial cells and podocytes [18,23], suggesting the pathological roles of the TWEAK/Fn14 system in the pathogenesis of glomerular injury. However, it is unknown how TWEAK contributes to the pathogenesis of IgAN. In the present study, we examined whether the levels of TWEAK are associated with histological findings and disease activity in patients with IgAN. In addition, we investigated the expression of TWEAK/Fn14 in the renal tissues of those patients and assessed the effect of TWEAK in glomerular mesangial cells and podocytes.

## Methods

### Patients and controls

This study included patients who had undergone renal biopsy in the Juntendo University Hospital, Tokyo, Japan, from January 2005 to March 2011. Although we recruited 236 patients with biopsy-proven IgAN during this period, 96 patients were not included because urine samples were not obtained or there was poor conservation of pathologic materials. Of the remaining 140 patients, 15 had insufficient clinical data and thus were excluded. Nine of the remaining 125 renal biopsy samples contained less than 8 glomeruli, and thus were excluded according to the Oxford classification [24,25]. In the end, 116 patients with IgAN were included in this study.

We enrolled 50 patients with non-IgAN kidney diseases, including 12 patients with minimal change disease (MCD), 12 patients with membranous nephropathy (MN), 18 patients with lupus nephritis (LN), and 8 patients with focal segmental glomerulosclerosis (FSGS) as disease controls. We also recruited 50 healthy subjects to serve as healthy controls.

Of the 116 patients with IgAN, 37 patients had follow-up data. They received steroid therapy (*n* = 8) or both steroid therapy and tonsillectomy (*n* = 29). The steroid therapy regimen consists of 0.5 g/day of methylprednisolone for 3 days, three times every 2 months, and patients were given oral prednisolone (0.5 mg/kg body weight) on alternate days for 6 months. The evaluation of therapeutic response of the IgAN patients was defined as follows [26]: clinical remission (CR, proteinuria <0.3 g/gCr with urinary sediment RBC <5 /HPF), partial remission (PR, proteinuria <0.3 g/gCr or urinary sediment RBC <5 /HPF).

This study was conducted according to the Declaration of Helsinki and was approved by Institutional Review Board of Juntendo University Hospital. Informed consent was obtained from all patients and healthy subjects.

### Sample collection

At the time of renal biopsy, all patients provided blood and freshly voided urine samples. The sera and supernatant from urine were separated by centrifugation, and were then stored in aliquots at −80°C prior to measurement. Urine samples from the 37 patients with follow-up were also collected and stored.

### Measurement of human serum and urinary TWEAK

The levels of serum TWEAK (sTWEAK) and uTWEAK were determined in duplicate with commercially available enzyme-linked immunosorbent assay (ELISA) kits (R&D Systems, Minneapolis, MN), following the manufacturer’s protocol. TWEAK assays were performed blindly, without knowledge of the patients’ disease status or activity.

### Measurement of other markers

Serum and urine measures from patients were determined in the clinical laboratory center in the Juntendo University Hospital. Levels of urinary protein excretion and uTWEAK were individually normalized to urinary creatinine levels. The estimated glomerular filtration rate (eGFR) was calculated using the Japanese eGFR equation [27].

### Pathologic analysis

All renal biopsy specimens were evaluated with immunofluorescence, light-, and electron microscopy. For light microscopy, paraffin sections were stained with hematoxylin-eosin (HE), periodic acid-Schiff (PAS), and periodic acid methenamine silver-Masson trichrome (PAM-MT). The Oxford classification was used to evaluate histologic findings of renal biopsy specimens of IgAN patients [24,25]. We also evaluated renal biopsy sections using the histological classification for IgAN in Japan [28], of which the prognosis classification is as follows: global sclerosis, segmental sclerosis, or cellular/fibrocellular/fibrous crescent were observed in <25% (Grade I), 25–49% (Grade II), 50–74% (Grade III) and ≥75% (Grade IV) of all glomeruli. The percentage of glomeruli with each glomerular lesion was analyzed. The histologic findings of each slide were evaluated by two nephrologists who did not know the details of the patients’ clinical data.

### Immunohistochemistry

Renal biopsies were performed according to the clinical needs of the patients in the Juntendo University Hospital. Immunohistochemistry was performed on formalin-fixed paraffin-embedded tissue sections. Briefly, sections (3 μm thick) were autoclaved at 121°C for 10 minutes in a 0.01 M citrate buffer (pH 6.0). To block endogenous biotin, the slides were treated with an avidin/biotin blocking kit (Vector Laboratories, Burlingame, CA) and treated with 0.3% H_2_O_2_ in methanol to inhibit endogenous peroxidase activity. The sections were visualized with first antibody for polyclonal goat anti-human TWEAK antibody (pAb) (R&D Systems), rabbit anti-human Fn14 pAb (Bioworld Technology, St. Louis Park, MN), or isotype-matched control IgG, and biotin-conjugated rabbit anti-goat IgG (DAKO, Carpinteria, CA) or goat anti-rabbit IgG (DAKO) by using avidin/biotin-peroxidase method.

### Cell culture

Conditionally immortalized mouse podocytes were kindly provided by Dr. K. Asanuma (Kyoto University, Kyoto, Japan) and Dr. P. Mundel (Massachusetts General Hospital, Boston, USA) and were cultured as previously described [29,30]. Podocytes were maintained in an RPMI 1640 medium (Sigma, Tokyo, Japan) supplemented with 10% FBS, penicillin/streptomycin (Life Technologies, CA), and 10 U/ml interferon-γ at 33°C. Podocytes were incubated at 37°C for 10–14 days to differentiate without interferon-γ.

Mouse mesangial cells (MMC, SV 40 MES-13) were obtained from the American Type Culture Collection (Manassas, Va). The cells were grown at 37°C in a 3:1 mixture of Dulbecco’s Modified Eagle’s and Ham’s F12 media (Sigma) supplemented with 5% FBS, and penicillin/streptomycin.

### Cell proliferation assay

MMC (5 × 10^3^ cells per well) were seeded in 96-well plates in duplicate with medium containing 0.1% FBS. Recombinant mouse TWEAK (R&D Systems) at 0–1000 ng/ml was added at the beginning of the experiment. After stimulation of MMC with TWEAK for 24 hours, cell proliferation ELISA using BrdU was performed with a colorimetric immunoassay kit (Roche Diagnostics, Mannheim, Germany) following the manufacturer’s instructions.

### Cytokine detection

The concentration of MCP-1 in supernatants of MMC culture was examined by a mouse MCP-1 ELISA kit (R&D Systems) according to the manufacturer’s instructions. Recombinant mouse TWEAK was used at 0, 10, and 100 ng/ml, for 3, 6, 12, and 24 hours.

### Wound healing assay

Wound healing assays were conducted as reported previously [30,31]. Differentiated wild-type podocytes (5 × 10^5^) were seeded overnight in six-well plates. Monolayers were scratched with a 200 μl pipette tip, washed with PBS and added to fresh medium with TWEAK (0–1000 ng/ml). The monolayers were photographed using a grid as a marker, and the wound width (μm) was measured at 0, 12 and 24 hours using BZ-II Measurement Module (Keyence, Osaka, Japan) with BZ Viewer™ (Keyence). Migratory rates were calculated as (A − B)/A × 100% or (A − C)/A × 100%, with A,B, and C reflecting the width of the wound at 0, 12, or 24 hours, respectively.

### Statistical analyses

Data were expressed as proportions, mean ± SD, or median (interquartile range [IQR]) as appropriate. Categorical variables were compared using the *χ*^2^ test. Continuous variables were compared using unpaired *t* test or Mann–Whitney *U* test as appropriate. We evaluated the differences in each biochemical parameter among the glomerular diseases by a one-way analysis of variance (ANOVA) followed by a multiple comparison analyses. Each multiple comparison analysis was performed with Tukey’s HSD (honest significance difference) mean separation test (parametric) or a Steel-Dwass test (nonparametric). Correlate variables were evaluated using Spearman’s rank correlation coefficient test. Statistical analyses were performed using JMP 9.0 statistical software (SAS Institute, Cary, NC) and GraphPad Prism version 6.0 software (GraphPad Software, San Diego, CA). A *P* value <0.05 was considered significant.

## Results

### Demographic and clinical characteristics of patients with IgAN and controls

The demographic and clinical characteristics of the IgAN patients and controls are summarized in Table 1. The mean age of the IgAN patients at diagnosis was 34.0 ± 10.9 years (range, 15–65 years). The MCD patients, the MN patients, and the LN patients excreted significantly more urinary protein than IgAN patients. The levels of uTWEAK in the IgAN patients (median: 94.3, IQR 65.1–147.1 pg/mgCr) did not differ from any other disease groups. In our cohort, however, the levels of uTWEAK in the patients with IgAN, LN, MN, FSGS, and MCD were significantly higher than in the healthy controls (*P* < 0.001, *P* = 0.001, *P* < 0.001, *P* = 0.005, and *P* = 0.001, respectively).

**Table 1:** **Demographic and clinical characteristics of IgAN patients, disease controls, and healthy controls**

**Characteristics**	**IgAN**	**LN**	**MN**	**FSGS**	**MCD**	**Healthy controls**	***P*** **value**
	**(** ***n*** **= 116)**	**(** ***n*** **= 18)**	**(** ***n*** **= 12)**	**(** ***n =*** **8)**	**(** ***n =*** **12)**	**(** ***n*** **= 50)**	
Age (years)	34.0 ± 10.9	33.0 ± 9.9	55.7 ± 9.5 ^A-E^	35.9 ± 13.1	31.8 ± 19.3	33.6 ± 5.5	<0.001
Men, *n* (%)	53 (45.7)	2 (11.1)	9 (75.0)	5 (62.5)	6 (50.0)	32 (64.0)	0.002
BMI (kg/m^2^)	21.7 ± 3.0	21.7 ± 4.5	23.5 ± 2.5	24.3 ± 5.3	23.3 ± 3.8	22.0 ± 2.0	0.08
Mean arterial pressure (mmHg)	83.8 ± 13.8	82.4 ± 16.3	87.3 ± 8.9	85.8 ± 10.1	79.1 ± 8.1	86.0 ± 11.0	0.62
eGFR (ml/min per 1.73 m^2^)	81.9 ± 29.0	82.8 ± 32.6	79.3 ± 29.4	76.8 ± 28.3	95.7 ± 23.7	N/A	0.56
CKD Stages 1/2/3/4/5 (KDOQI) (%)^a^	32/47/20/1/0	44/39/11/0/6	25/50/25/0/0	38/50/0/13/0	50/42/8/0/0	-	0.13
Urinary protein excretion (g/gCr)	0.61 ± 0.88	2.22 ± 2.62 ^F^	3.76 ± 2.97 ^G^	1.70 ± 1.91	5.46 ± 4.35 ^H,I,J^	N/A	<0.001
uTWEAK (pg/mgCr, median, IQR)	94.3 (65.1, 147.1)^K^	130.8 (70.2, 163.8)^L^	151.9 (98.8, 285.1)^M^	168.9 (107.8, 304.7)^N^	180.6 (92.8, 331.8)^O^	64.2 (32.2, 84.9)	<0.001

Abbreviations: BMI, body mass index; eGFR, estimated glomerular filtration rate; uTWEAK, urinary TWEAK; IQR, interquartile range; IgAN, IgA nephropathy; LN, lupus nephritis; MN, membranous nephropathy; FSGS, focal segmental glomerulosclerosis; MCD, minimal change disease; NA, not available; ANOVA, analysis of variance; HSD, honest significant difference.

^a^CKD stage 1, 2, 3, 4, and 5 were divided by eGFR ≥90, 60–89, 30–59, 15–29, and <15, respectively.

Data are expressed as proportions, mean ± SD, or median (interquartile range [IQR]) as appropriate. Differences among the groups were analyzed by a one-way ANOVA. The multiple comparisons for age, BMI, mean arterial pressure, eGFR, and urinary protein excretion were performed by Tukey’s HSD mean separation tests. A nonparametric Steel-Dwass test was used for uTWEAK. Differences between the disease groups in gender and CKD stages were determined by *χ* ^2^ tests. A: *P* < 0.001 vs. IgAN; B: *P* < 0.001 vs. LN; C: *P* < 0.001 vs. FSGS; D: *P* < 0.001 vs. MCD; E: *P* < 0.001 vs. healthy controls; F: *P* = 0.001 vs. IgAN; G: *P* < 0.001 vs. IgAN; H: *P* < 0.001 vs. IgAN; I: *P* < 0.001 vs. LN; J: *P* < 0.001 vs. FSGS; K: *P* < 0.001 vs. healthy controls; L: *P* = 0.001 vs. healthy controls; M: *P* < 0.001 vs. healthy controls; N: *P* = 0.005 vs. healthy controls; O: *P* = 0.001 vs. healthy controls.

**Figure 1:**
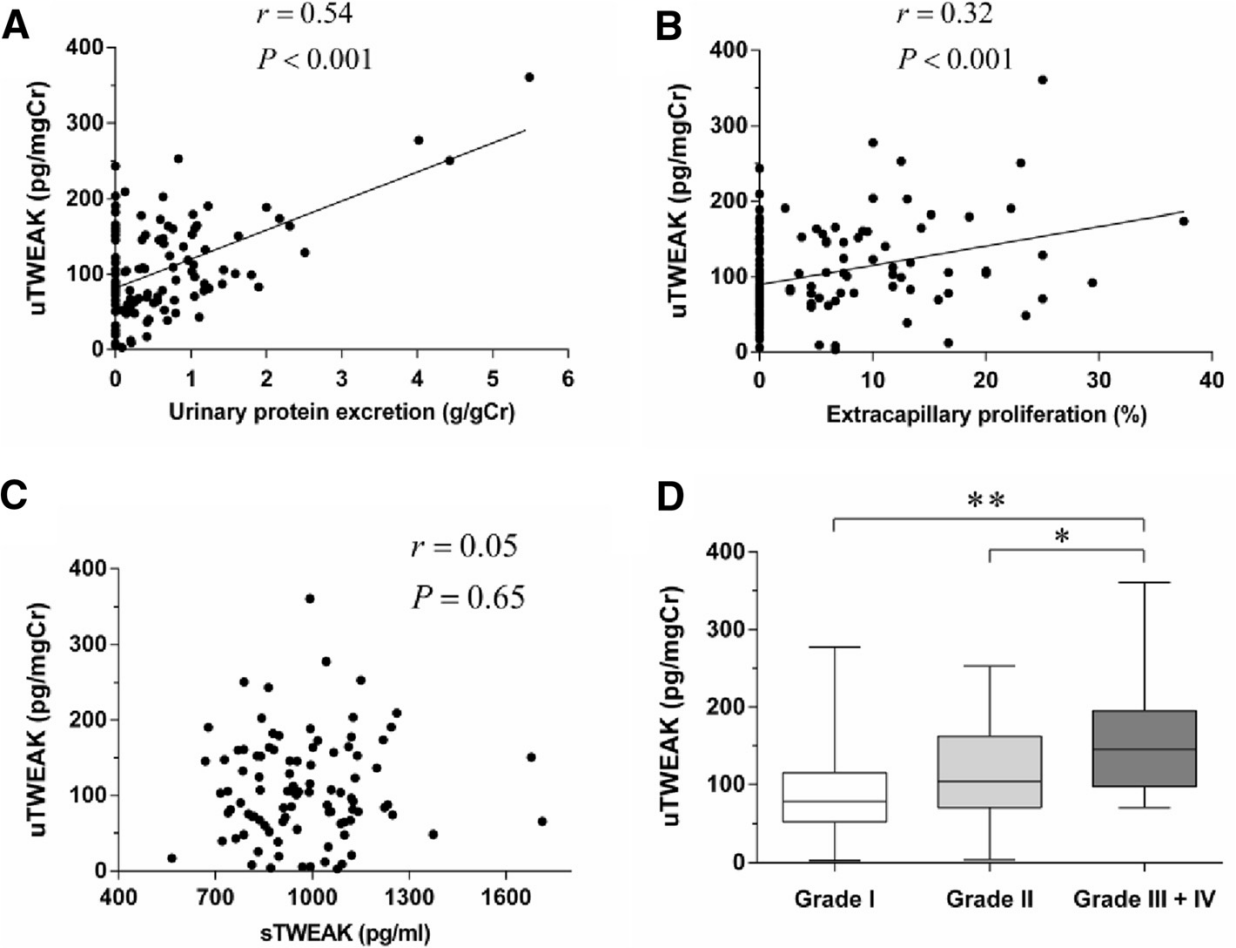
**Relationship between the levels of uTWEAK and clinical and histopathologic characteristics in patients with IgAN.** The levels uTWEAK at the time of renal biopsy in 116 IgAN patients showed significantly correlations with: **(A)** urinary protein excretion, *r* = 0.54, *P* < 0.001; and **(B)** extracapillary proliferation, *r* = 0.32, *P* < 0.001. **(C)** There was no significant association between the levels of serum TWEAK (sTWEAK) and uTWEAK (r = 0.05, *P* = 0.65). **(D)** Box plots of the levels of uTWEAK with different histological grades in patients with IgAN. The level of uTWEAK in Grade III + IV (*n* = 12, median: 145.7, IQR 97.5-195.6 pg/mgCr) was significantly higher than those in Grade I (*n* = 63, median: 78.6, IQR 52.3-115.7 pg/mgCr; **P < 0.001) or Grade II (*n* = 41, median: 103.7, IQR 70.9–162.5 pg/mgCr; *P < 0.05). The lines in the box plots and the error bars are median and 10–90 percentiles.

The levels of uTWEAK were significantly correlated with urinary protein levels in IgAN patients (r = 0.54, *P* < 0.001, Figure 1A), in MN patients (r = 0.77, *P* = 0.003), and in MCD patients (r = 0.76, *P* = 0.004). No correlation was detected between the levels of uTWEAK and urinary protein excretion in LN patients (r = 0.17, *P* = 0.51) and FSGS patients (r = 0.20, *P* = 0.65). There was a significant correlation between the levels of uTWEAK and the extracapillary proliferation in IgAN patients (Figure 1B).

### Clinical and histopathologic characteristics in subgroups of IgAN patients

The IgAN patients were next divided into three equal groups according to the tertiles of the uTWEAK distribution: Group 1, uTWEAK levels <72.3 pg/mgCr; Group 2, uTWEAK levels between 72.3 and 123.0 pg/mgCr; and Group 3, uTWEAK levels >123.0 pg/mgCr (Table 2). The urinary protein excretion, degree of interstitial fibrosis and extracapillary proliferation in the IgAN patients of Group 3 were significantly higher than the IgAN patients of Group 1 (*P* < 0.001, *P* = 0.009, and *P* = 0.02, respectively). Among the cases, we observed no significant association between the levels of serum TWEAK (sTWEAK) and the clinical and histological parameters, including uTWEAK (Figure 1C).

**Table 2:** **Clinical and histopathologic characteristics in subgroups of IgAN patients defined by tertiles of uTWEAK**

**Characteristics**	**IgAN Group 1 (** ***n*** **= 39)**	**IgAN Group 2 (** ***n*** **= 39)**	**IgAN Group 3 (** ***n*** **= 38)**	***P*** **value**
	**pg/mgCr, median (IQR)**	**pg/mgCr, median (IQR)**	**pg/mgCr, median (IQR)**	
	**50.7 (21.1, 65.3)**	**96.4 (81.2, 105.7)**	**163.8 (147.1, 190.6)**	
**Clinical**				
Age (years)	33.2 ± 10.2	32.9 ± 10.9	36.1 ± 11.6	0.39
Men, *n* (%)	16 (41.0)	25 (64)	12 (32)	0.01
BMI (kg/m^2^)	21.4 ± 2.7	22.4 ± 2.9	21.4 ± 3.3	0.22
Mean arterial pressure (mmHg)	82.8 ± 11.2	84.1 ± 19.2	84.6 ± 9.5	0.84
eGFR (ml/min per 1.73 m^2^)	83.3 ± 25.8	82.4 ± 35.2	80.0 ± 25.8	0.88
CKD Stages 1, 2, 3, and 4 (KDOQI) (%)				
Stage 1: >90	15 (38.5)	9 (23.7)	13 (34.2)	0.35
Stage 2: 60-89	16 (41.0)	23 (60.5)	15 (39.5)	
Stage 3: 30-59	7 (17.9)	6 (15.8)	10 (26.3)	
Stage 4: 15-29	1 (2.6)	0 (0)	0 (0)	
Urinary protein excretion (g/gCr)	0.28 ± 0.30	0.51 ± 0.60	1.03 ± 1.28 ^A,B^	<0.001
class 0: <0.30	24 (61.5)	20 (51.3)	11 (28.9)	0.004
class 1: 0.30-0.99	13 (33.3)	8 (20.5)	13 (34.2)	
class 2: 1.00-2.99	2 (5.2)	11 (28.2)	11 (28.9)	
class 3: ≥3.00	0 (0)	0 (0)	3 (7.9)	
sTWEAK (pg/ml, median, IQR)	921.4 (828.8, 1089.1)	957.1 (874.3, 1103.7)	941.5 (836.4, 1124.6)	0.95
**Histopathologic** mean ± SD (range)				
Total glomerular number	18.2 ± 7.9 (8–42)	19.6 ± 7.5 (8–37)	18.7 ± 8.0 (8–44)	0.74
Global glomerular sclerosis (%)	7.6 ± 11.0 (0–50.0)	11.0 ± 13.5 (0–62.5)	13.7 ± 13.3 (0–44.4)	0.11
Mesangial hypercellularity score	0.47 ± 0.29 (0.09-1.3)	0.57 ± 0.30 (0.1-1.6)	0.61 ± 0.25 (0.13-1.1)	0.12
Endocapillary hypercellularity (%)	3.0 ± 5.8 (0–26.7)	1.6 ± 2.9 (0–13.3)	3.7 ± 5.6 (0–20.0)	0.15
Segmental glomerulosclerosis (%)	4.0 ± 8.1 (0–40.0)	5.5 ± 7.4 (0–37.5)	6.6 ± 9.1 (0–33.3)	0.37
Interstitial fibrosis (%)	14.3 ± 5.7 (6.3-28.0)	15.4 ± 5.6 (3.8-32.8)	18.6 ± 7.1 (7.9-39.7) ^C^	0.01
Extracapillary proliferation (%)	3.6 ± 6.6 (0–25.0)	5.9 ± 7.5 (0–29.4)	8.5 ± 8.9 (0–37.5) ^D^	0.02
Histological-grade I/II/III/IV (%)^a^	69/28/0/3	59/33/8/0	34/44/16/5	0.03

Abbreviations: BMI, body mass index; eGFR, estimated glomerular filtration rate; uTWEAK, urinary TWEAK; sTWEAK: serum TWEAK; IQR, interquartile range; ANOVA, analysis of variance; HSD, honest significant difference.

^a^Histological-grade was classified according to the histological classification for IgAN in Japan [28].

Differences among the groups were analyzed by a one-way ANOVA. The multiple comparisons for urinary protein excretion, interstitial fibrosis, and extracapillary proliferation were performed by Tukey’s HSD mean separation tests. Categorical data were determined by *χ* ^2^ tests. A: *P* < 0.001 vs. Group 1; B: *P* = 0.02 vs. Group 2; C: *P* = 0.009 vs. Group 1; D: *P* = 0.02 vs. Group 1.

### Association of uTWEAK levels with histologic characteristics

The distribution by histological Grades I, II, III, and IV was 54.3, 35.3, 7.8, and 2.6%, respectively. The level of uTWEAK in Grade III + IV was significantly higher than those in Grades I or II (Figure 1D). In the Oxford classification, the levels of uTWEAK in IgAN patients with M1, En1, S1, and T1, 2 were relatively higher than those with M0, En0, S0, and T0, although a significant difference was not detected (Table 3).

**Table 3:** **Renal biopsy section evaluated with the Oxford classification (MEST)**

**Oxford classification**		**IgAN patients,**	**uTWEAK**	***P*** **value** ^**a**^
		***n*** **(%)**	**(pg/mgCr, median, IQR)**	
M	0	59 (50.9)	92.1 (64.5, 136.7)	0.49
	1	57 (49.1)	96.4 (66.6, 154.8)	
E	0	77 (66.4)	87.3 (64.7, 141.1)	0.18
	1	39 (33.6)	106.0 (67.8, 160.1)	
S	0	62 (53.4)	81.2 (59.9, 145.5)	0.20
	1	54 (46.6)	103.5 (73.3, 151.2)	
T	0	106 (91.4)	91.1 (64.9, 145.5)	0.21
	1, 2	10 (8.6)	150.9 (63.9, 184.7)	

Abbreviations: uTWEAK, urinary TWEAK Mesangial hypercellularity score of ≤0.5 (M0) or >0.5 (M1), absence (E0) or presence (E1) of endocapillary hypercellularity, absence (S0) or presence (S1) of segmental glomerulosclerosis, and tubular atrophy/interstitial fibrosis of 0–25% (T0), 26–50% (T1), and >50% (T2).

^a^Mann-Whitney *U* test.

Both univariate and multivariate logistic analyses were performed to evaluate the impact of uTWEAK on the histologic lesions (Table 4). Interstitial fibrosis and extracapillary proliferation were statistically significant factors that were associated with the levels of uTWEAK. In a multivariate analysis, extracapillary proliferation was a significant independent factor that impacted the levels of uTWEAK.

**Table 4:** **Univariate and multivariate regression analyses of the pathologic factors that associate with uTWEAK in IgAN**

**Variable**	**Univariate analysis**		**Multivariate analysis**	
	***r***	***P*** **value**	***t***	***P*** **value**
Global glomerular sclerosis (%)	0.20	0.03	1.28	0.20
Mesangial hypercellularity score	0.14	0.13	0.27	0.79
Endocapillary hypercellularity (%)	0.18	0.05	1.49	0.14
Segmental glomerulosclerosis (%)	0.11	0.23	0.45	0.65
Interstitial fibrosis (%)	0.24	0.01	1.30	0.20
Extracapillary proliferation (%)	0.32	<0.001	2.72	0.007

### The changes in the levels of uTWEAK at renal biopsy and during follow-up

**Figure 2:**
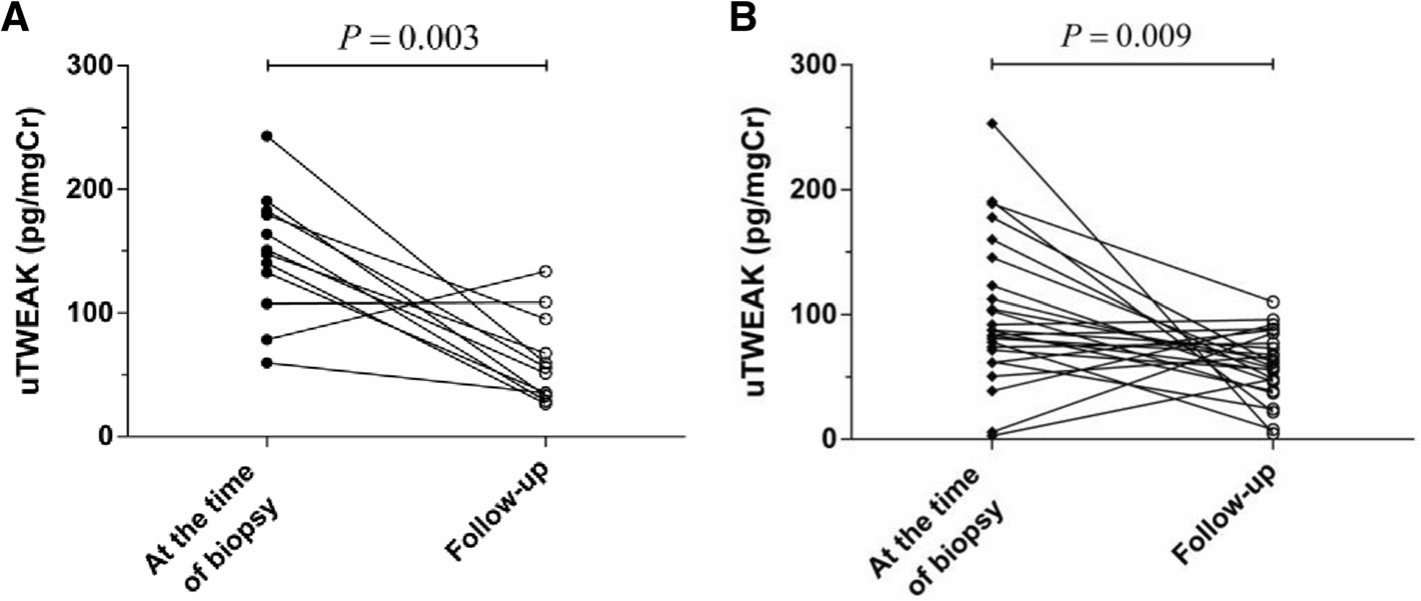
**The changes in the levels of uTWEAK in patients with IgAN during follow-up. (A)** Patients with clinical remission (*n* = 12): the levels of uTWEAK at the time of biopsy (median: 149.2, IQR 113.8–181.8 pg/mgCr) decreased significantly compared with those of therapy-induced clinical remission (median: 53.8, IQR 33.6–88.5 pg/mgCr; *P* = 0.003). **(B)** Patients with partial remission (*n* = 25): the levels of uTWEAK at the time of biopsy (median: 87.3, IQR 67.1–134.3 pg/mgCr) also decreased compared with those of therapy-induced partial remission (median: 58.4, IQR 42.6-81.3 pg/mgCr; *P* = 0.009). Wilcoxon signed-rank test.

During follow-up with a median duration of 36 (IQR 24–40) weeks, we obtained urine samples from 37 patients with therapeutic responses. There were 12 patients achieving CR, 25 patients achieving PR, meaning that all patients responded to the treatment. At diagnosis the levels of uTWEAK in the patients with CR were significantly higher than those in PR (*P* < 0.05). In a comparison of the levels of uTWEAK at diagnosis with that of follow-up, the levels of uTWEAK in patients with CR and PR decreased significantly (Figure 2, A and B).

### Expression of TWEAK and Fn14 in renal biopsies from IgAN patients

**Figure 3:**
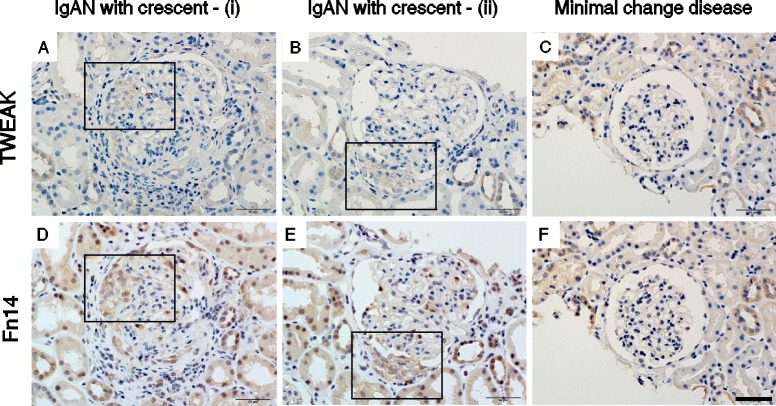
**Expression of TWEAK and Fn14 in renal biopsies from IgAN patients was examine.** Immunohistochemistry for both TWEAK **(A, B)** and Fn14 **(D, E)** were detected in glomerular crescents (the areas delineated by the squares). In the controls [renal biopsies from patients with minimal change disease **(C, F)**], there was very slight staining for TWEAK and Fn14 in glomeruli, while intense staining was observed in renal tubular cells. Scale bar = 50 μm.

We investigated the localization of TWEAK in the renal biopsies from IgAN patients by immunohistochemistry. The expression of both TWEAK and Fn14 were increased in renal tubular cells in IgAN patients (Figure 3, A, B, D, and E). In the IgAN patients with glomerular crescents, TWEAK and Fn14 were detected in glomerular tufts and the crescents. In the controls (renal biopsies from patients with MCD), two images of the same glomeruli showed very slight staining for TWEAK and Fn14 in glomeruli, while intense staining was observed in renal tubular cells (Figure 3, C and F). TWEAK and Fn14 were also detected in the glomeruli of other crescentic glomerulonephritis (GN), including ANCA-associated renal vasculitis (data not shown).

### TWEAK regulates mesangial cell proliferation and podocyte migration

**Figure 4:**
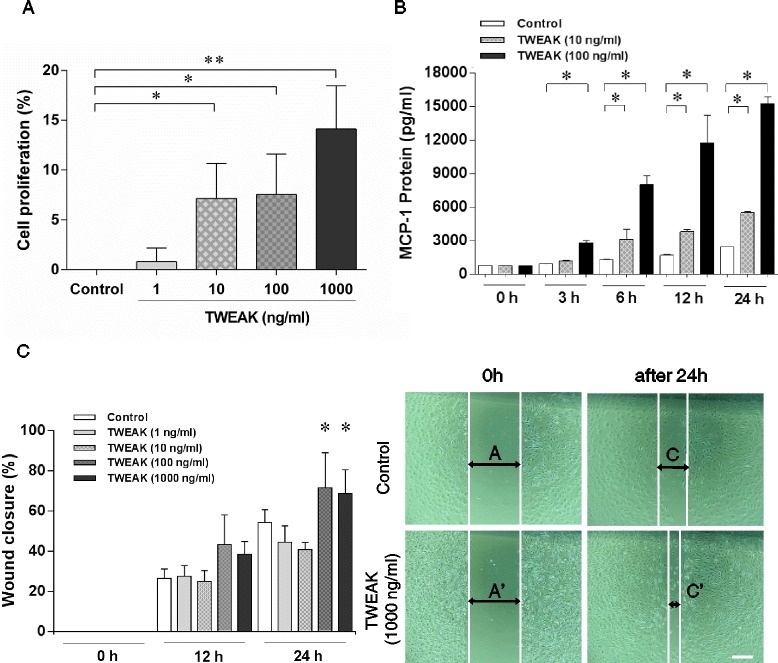
**TWEAK actions on renal cells** ***in vitro*** **. (A)** TWEAK modulates mesangial cells proliferation. The proliferation of MMC is significantly increased following stimulation with TWEAK (10–1000 ng/ml) for a 24-hour incubation. Data are expressed as mean ± SD of five independent experiments. **P < 0.001 vs. control; *P < 0.05 vs. control. **(B)** MCP-1 secretion in response to TWEAK stimulation is dose and time dependent. MCP-1 protein levels in TWEAK stimulated MMC supernatants are shown. Data are expressed as mean ± SD of three independent experiments. *P < 0.05, TWEAK at 10, 100 ng/ml vs. control. **(C)** TWEAK stimulates podocyte motility as evaluated by wound healing assay. After the scraping of the podocyte cell layer, cells have started to migrate into the wound track. The wound closures were significantly enhanced in the presence of TWEAK (100, 1000 ng/ml) at 24 hours. Compared with the control, TWEAK enhanced directed cell migration in differentiated podocytes. Data are expressed as mean ± SD of five independent experiments. *P < 0.05, TWEAK at 100, 1000 ng/ml vs. control 24 hours. Scale bar = 200 μm.

To confirm whether TWEAK may affect the proliferation or apoptosis of kidney cells, we performed a cell proliferation assay. The proliferation of MMC was significantly increased under the stimulation of TWEAK (10–1000 ng/ml), with the highest effect at 1000 ng/ml (Figure 4A). We examined TWEAK-induced MCP-1 expression in MMC. Protein expression was increased in both the dose of TWEAK and time-dependent manners (Figure 4B).

Next, we analyzed whether TWEAK has a role in the motility of podocytes. In the wound healing assay, as compared with the control at 24 hours, TWEAK (100, 1000 ng/ml) significantly enhanced cell motility in differentiated podocytes (Figure 4C).

## Discussion

In the present study, we showed that the urine excretion of soluble TWEAK is associated with clinicopathological findings in patients with IgAN. Notably, we also demonstrated that TWEAK significantly enhanced cell motility in podocytes and in the proliferation of mesangial cells, known to be an important feature of the histopathology of IgAN. Mesangial cells and podocytes constitutively express TWEAK and Fn14 [18,23]. Most cases of proteinuria are associated with the effacement of podocyte foot processes, which represents podocyte dynamics *in vivo* or the motility of podocytes [13,30]. Podocytes stay attached to the GBM, but an altered cell motility of podocytes results in foot processes effacement and proteinuria. Since TWEAK enhanced podocyte motility, Fn14 activation in podocytes may associate with the development of proteinuria. In fact, we confirmed that the levels of uTWEAK were significantly correlated with urinary protein excretion not only in IgAN patients, but also in MCD and MN patients. However, no correlation was observed between the levels of uTWEAK and the degree of proteinuria in LN patients, as seen in a previous study [8]. Therefore, LN with proteinuria may be affected by additional other factors or mediators.

The underlying mechanisms as to why TWEAK relates to the development of proteinuria are unknown. However, experimental studies, using pharmacological or genetic approaches, have clearly shown that TWEAK activates Fn14 following renal inflammation [22,32]. TWEAK activates the canonical nuclear factor-kB (NF-κB) pathway to induce the expression of both soluble and membrane-bound inflammatory chemokines, including MCP-1, RANTES and CXCL16 [18,20,23]. The present results showed that TWEAK modulates cell proliferation and MCP-1 production in glomerular mesangial cells in a dose-dependent manner. Recently, it has been demonstrated that TWEAK activates NF-κB and increases MCP-1 expression in podocytes [33]. Podocyte-derived MCP-1 could activate the cysteine-cysteine chemokine receptor 2 (CCR2) in podocytes in a autocrine or paracrine manner. Furthermore, in podocytes MCP-1 has been reported to promote motility [34,35], actin cytoskeleton rearrangement [35], and decreased nephrin expression [36]. Accordingly, these data may indirectly support our findings and suggest that the TWEAK/Fn14 pathway increases inflammatory settings and thus further enhances cell motility in podocytes leading to proteinuria.

The Oxford classification of IgAN identified pathological variables, the MEST score was associated closely with kidney failure, which was also validated in previous studies [37-40]. A recent study from Japan reported that a histological classification of IgAN could identify the risk of progression to ESRD [28]. Multivariate logistic analysis showed that the risk of progression to ESRD was higher in histological Grades II, III, and IV than in histological Grade I. Our results confirmed that the levels of uTWEAK increased in parallel with the Japanese histological grade. However, we did not detect any significant relationship between uTWEAK and each parameter in the Oxford classification. Extremely mild and severe cases were excluded in this classification [41]. It is possible that our study cohort consisted of populations that were lower in proteinuria and higher in eGFR as compared with that of other studies [37-39] as we could not detect differences in each histologic parameter.

In a multivariate analysis, we found that extracapillary proliferation was a significant independent factor that was associated with the levels of uTWEAK. To confirm whether these findings are relevant to pathophysiological conditions, we evaluated both TWEAK and Fn14 expression in renal biopsies. In the control kidneys, we observed a very slight staining for TWEAK and Fn14 in glomeruli. By contrast, TWEAK and Fn14 were detected in glomerular crescents in the IgAN patients with crescents, which is consistent with the clinical relationship between uTWEAK and extracapillary proliferation. Although glomerular crescents are recognized as a heterogeneous composition of cells and matrix, recent evidence suggests the presence of podocytes in these structures [42-44]. The dedifferentiation of podocytes leads to podocyte proliferation within Bowman’s space and the collapse of glomerular tufts [13]. Further proliferation of podocytes and parietal cells result in the formation of crescents [13,45]. Moreover, the potential contribution of proinflammatory cytokines and macrophages during the effector phase of crescentic GN has been indicated [46]. These findings, together with TWEAK-induced cell motility in podocytes and TWEAK and Fn14 expression in crescents of IgAN suggest that TWEAK/Fn14 may be involved in the podocyte alterations and subsequent crescent formation.

The present study showed that the levels of uTWEAK in IgAN patients significantly decreased according to the treatment responses. Urinary biomarkers are attractive candidates for accurately reflecting the activity of kidney injury. Our findings indicate that uTWEAK may be useful as a biomarker to predict the histologic findings, especially crescent formation and disease activity in IgAN.

Although we could not identify which cells are responsible for the TWEAK production, our results indicate that TWEAK locally production and the subsequent inflammatory responses may be involved in podocytes, mesangial cells, and infiltration of macrophages in the kidney. It appears that there are different causes of increased uTWEAK levels between the IgAN patients and the MCD patients. In the MCD patients, podocyte injuries and loss of GBM charge would result in the massive leakages of TWEAK, and probably be the main cause of the high levels of uTWEAK. On the other hand, the production of TWEAK in the IgAN patients may be associated with the leakages and injured glomerular cells such as podocytes and mesangial cells.

This study has several limitations. First, the levels of uTWEAK do not clearly distinguish the patient groups, there seems to be substantial overlap between IgAN and CKD. Second, a decrease in uTWEAK is shown in IgAN patients who receive steroid therapy, we were not able to analyze the changes of uTWEAK in those patients who did not receive immunosuppressive therapies because urine samples were not obtained. Finally, this study was performed only in Juntendo University Hospital, and the number of patients was small. Further studies are required to elucidate the mechanisms underlying TWEAK/Fn14 signaling and its role in pathophysiology of patients with IgAN.

## Conclusion

In conclusion, the relationship between the levels of uTWEAK and clinicopathological findings observed in this study suggests that TWEAK/Fn14 system affects crescent formation and proteinuria in patients with IgAN.

## Abbreviations

TWEAK: TNF-like weak inducer of apoptosis
Fn14: Fibroblast growth factor-inducible-14
uTWEAK: Urinary TWEAK
CKD: Chronic kidney disease
IgAN: IgA nephropathy
ESRD: End-stage renal disease
GBM: Glomerular basement membrane
MCP-1: Monocyte chemoattractant protein-1
IL: Interleukin
MCD: Minimal change disease
MN: Membranous nephropathy
LN: Lupus nephritis
FSGS: Focal segmental glomerulosclerosis
CR: Clinical remission
PR: Partial remission
sTWEAK: Serum TWEAK
ELISA: Enzyme-linked immunosorbent assay
eGFR: Estimated glomerular filtration rate
IQR: Interquartile range
ANOVA: Analysis of variance
GN: Glomerulonephritis
NF-κB: Nuclear factor-kB
